# Efficacy of brain natriuretic peptide *vs*. nicorandil in preventing contrast-induced nephropathy: a network meta-analysis

**DOI:** 10.7717/peerj.12975

**Published:** 2022-02-23

**Authors:** Ziwei Mei, Songmei Luo, Peipei Chen, Qiankun Zhang, Limei Zhou, Chaoyong Zhu, Hong Zhu, Lie Jin

**Affiliations:** 1 Pharmacy Department, Lishui Municipal Central Hospital, Lishui, Zhejiang, China; 2 Nephrology Department, Lishui Municipal Central Hospital, Lishui, Zhejiang, China

**Keywords:** Contrast-induced nephropathy, Brain natriuretic peptide, Nicorandil, Meta-analysis

## Abstract

This study aimed to conduct a network meta-analysis (NMA) to compare the efficacy of brain natriuretic peptide (BNP) vs nicorandil for preventing contrast-induced nephropathy (CIN). Databases of Pubmed, Cochrane, Embase, Web of Science were searched by keywords for eligible studies of randomized controlled trials investigating different agents (BNP, nicorandil, nitroglycerin, intravenous saline) for preventing CIN. The outcomes included a change in serum creatinine level at 48 h and the incidence of CIN after percutaneous coronary intervention (PCI) or coronary angiography (CAG). A total of 13 studies with 3,462 patients were included. Compared with intravenous saline alone, except for nitroglycerin (odds ratio [OR]: 1.02, 95% CI [0.36–2.88]), the other drugs significantly reduced the CIN incidence with OR of 0.35 (95% CI [0.24–0.51]) for BNP, 0.52 (0.29, 0.94) for usual-dose nicorandil, 0.28 (0.19, 0.43) for double-dose nicorandil. BNP and double-dose nicorandil significantly decreased the change of serum creatinine (SCr) levels with mean difference (MD) of −6.98, (−10.01, −3.95) for BNP, −8.78, (−11.63, −5.93) for double-dose nicorandil. No significant differences were observed in the change of SCr levels for nitroglycerin (−4.97, [−11.46, 1.52]) and usual-dose nicorandil (−2.32, [−5.52, 0.89]) compared with intravenous saline alone. For double-dose nicorandil, the CIN incidence and the change of SCr level in group of 4–5 days treatment course were more than group of less than or equal to 24 h treatment course (OR of 1.48, [0.63–3.46] and MD of 2.48, [−1.96, 6.91]). In conclusion, BNP and double-dose nicorandil can have effects on preventing the incidence of CIN and double-dose nicorandil performed better than BNP. In double-dose nicorandil groups, a course of less than or equal to 24 h before and after procedure performed with better efficacy than a course of 4–5 days.

## Introduction

PCI or CAG is a common method for coronary heart disease treatment and diagnosis. However, the application of contrast agents for patients undergoing CAG or PCI usually induces CIN. CIN refers to abrupt damage in renal function after the administration of contrast agents (Rear, Bell & Hausenloy, 2016; Owen et al., 2014; Grossman et al., 2017a). CIN is a serious complication featured by the deterioration of renal function which may lead to water-sodium retention aggravating heart failure and drug accumulation increasing adverse drug reaction (Tepel, Aspelin & Lameire, 2006). In the long term, it will induce damage to cardiovascular system and digestive system.

With the increased application of contrast agents for radiation diagnosis and interventional therapy, the rate of CIN continues to rise. It has been reported that the incidence of CIN varies from 2% to 50%, it likely more occurred in patients with risk factors, such as pre-existing renal impairment, diabetes mellitus, congestive heart failure, advanced age, and hypertension (Chyou et al., 2015). CIN is the third most common cause of hospital-acquired acute kidney injury prolonging hospitalization and increasing some poor outcomes, such as dialysis and cardiovascular disease (Ali-Hasan-Al-Saegh et al., 2017; Uzunhasan et al., 2017; Grossman et al., 2017b). Therefore, CIN has become one of the important issues affecting the survival and prognosis of patients.

Nowadays, there is no effective method to treat CIN, therefore more and more studies have been conducted to explore methods for preventing the occurrence of CIN (Subramaniam et al., 2016; Marenzi et al., 2015). A large number of randomized controlled trials (RCTs) have demonstrated pharmacological drugs could prevent the incidence of CIN. These years, prostaglandin analogues and BNP analogues are applied to prevent the CIN in the PCI and CAG. Some RCTs showed BNP and nicorandil interventions could reduce the incidence of CIN and SCr levels in the PCI and CAG (Wei et al., 2016a; Pranata et al., 2020). However, there was no study evaluating and comparing the efficacy of BNP and nicorandil on preventing CIN. This study conducted an NMA of RCTs to, directly and indirectly, compare the efficacy of BNP vs nicorandil for preventing CIN in PCI or CAG.

## Methods

The protocol of this NMA has been registered on the International Prospective Register of Systematic Review with a registration number CRD42021278424. We reported this NMA based on the Preferred Reporting Items for Systematic Reviews and Meta-Analyses (PRISMA) statement for NMA.

### Data sources and searches

Two reviewers (MZW and ZQK) independently searched the literature and disagreements were resolved by consensus-based discussion. We searched extensive literature from Pubmed, Cochrane, Embase, Web of Science, and clinicaltrials.gov databases. The deadline for publication for inclusion in the meta-analysis was August 2021. Our search terms and search strategy were ((nicorandil) OR (brain natriuretic peptide)) AND ((coronary angiography) OR (percutaneous coronary intervention)); (contrast-induced nephropathy) AND ((nicorandil) OR (brain natriuretic peptide)). In addition, references included to related meta-analysis were viewed as potential studies.

### Study selection

The final studies were selected by the following inclusion criteria: (1) full-RCTs; (2) evaluating the efficacy of CIN prevention; (3) all patients following PCI or CAG; (4) hydration is the co-intervention in the treatment and control groups; (5) reported sufficient data and at least one of the following outcomes: the incidence of CIN, SCr levels.

Studies were excluded according to the following features: (1) non-RCTs; (2) duplicate publication; (3) animal studies; (4) lacking data about the incidence of CIN and serum creatinine level.

### Endpoint

The primary outcome was CIN defined by an increase in SCr of >0.5 mg/dL or >25% from baseline within 48 h after PCI or CAG, the definition of CIN reported by the included study was accepted. The secondary endpoint was the SCr level change, before and after the procedure. If the SCr value was reported at multiple time points, we extracted it at 48 h after the procedure.

### Data extraction and quality assessment

Three reviewers (ZLM, CPP, and ZCY) independently extracted data from original trial reports in a standardized form. By discussion with a third reviewer (JL), discrepancies were settled. The characteristics of the enrolled studies in each group including first author, publication date, country, sample size, baseline characteristics of the patients, the incidence of CIN, SCr level were extracted. Each included study was assessed by the risk of bias evaluated tool from the Cochrane Handbook for Randomized Controlled Trials. This assessment was completed independently by two investigators (MZW and ZH) and disagreements were discussed with a third reviewer and resolved through consensus.

### Data analysis

We applied network meta-analysis to estimate the treatment effect of pharmacological interventions by the OR for the incidence of CIN and MD for SCr level at 48 h after the procedure with a 95%CI. The treatment hierarchy was summarized and reported according to surface under the cumulative ranking curve (SUCRA) and mean ranks. SUCRA was presented as a percentage and used to determine the probability of a treatment being the most effective, without uncertainty on the outcome. The higher probability viewed as the best intervention was the larger surface area under the curve.

Inconsistency was assessed by global inconsistency, loop-specific and node-splitting approach between direct and indirect evidence. In global inconsistency, *P* > 0.05 was considered there was no statistical significance about heterogeneity among the evidence. For the loop-specific approach, the extent of bias and inconsistency was evaluated by the inconsistency factor (IF). When the IF with a 95%CI included 0, it demonstrated the estimates of treatment effects from direct and indirect evidence are in agreement. For the node-splitting approach, the evidence about certain comparisons was separated into direct and indirect evidence and the result was reported by *P*-value, *P* > 0.05 indicated there is no inconsistency. Statistical analysis was performed by STATA 15.0 (Stata Corporation, College Station, Texas, USA).

## Results

### Study characteristics

**Figure 1:**
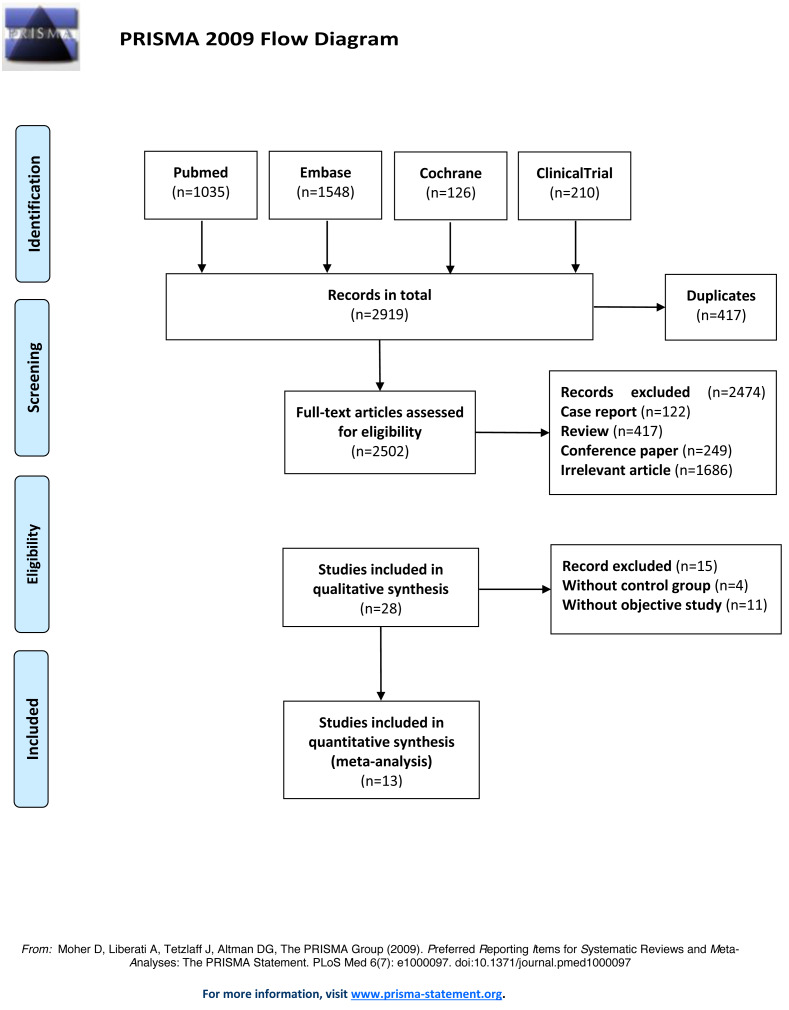
Flow diagram of literature search and selection. The flow diagram was depicted following the guideline of Preferred Reporting Items for Systematic Reviews and Meta-Analyses (PRISMA).

Thirteen RCTs studies were included for analysis after removing the duplicate studies, reviews, non-RCTs, and irrelevant content. The literature search process was shown in Fig. 1. The publication year of the included studies ranged from 2014 to 2019. 3,462 participants have included totally and female participants accounted for 31.43%. The sample sizes ranged from 128 to 1,000. The information and baseline characteristic of the studies were provided in Tables 1 and 2. The 13 RCTs contained the following comparisons: BNP vs hydration (*n* = 4), BNP vs nitroglycerin (*n* = 1), nicorandil vs hydration (*n* = 8).

### Quality of the included studies

Most of the studies were judged to be at low risk of bias for 6 domains (Fan et al., 2016a; Xing et al., 2016; Zeng et al., 2019; Zhang et al., 2010; Zhang et al., 2020b), according to the Cochrane Collaboration’s tool. 8 studies were judged to be at high risk of bias for they were not blinded (Fan et al., 2019; Iranirad et al., 2017; Ko et al., 2013; Liu et al., 2014; Liu et al., 2016; Zhang et al., 2020a; Nawa et al., 2015; Sun et al., 2015). One study was judged to be at high risk of bias for participants were randomized according to the participating centers and the severity of the renal dysfunction (estimated Glomerular Filtration Rate (eGFR) ≤40 or >40 mL/min) (Ko et al., 2013). The risk of bias assessment of the trials included in this study was presented in Fig. S1.

### Network meta-analysis results

**Figure 2:**
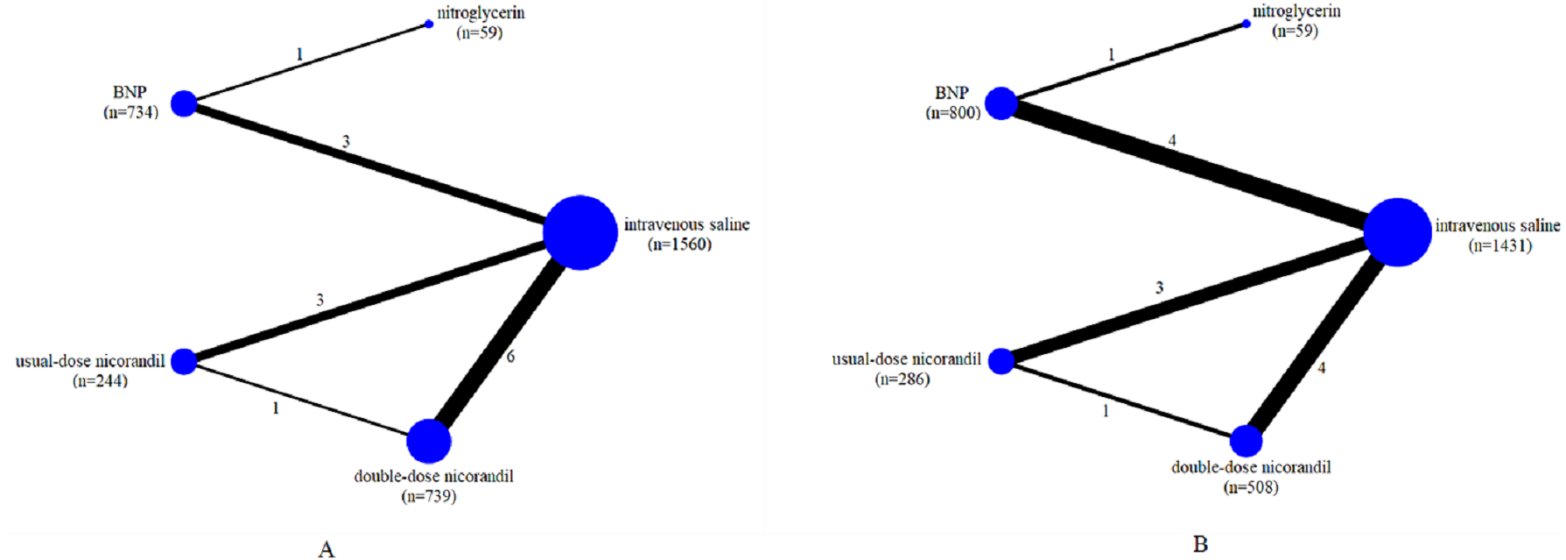
Network of all the drugs included in the analysis. (A) Network of all included agents for decreasing the occurrence of CIN. (B) Network of all included agents for the efficacy of reducing the change of SCr levels. Nodes present the comparison among treatments. The width of the lines and the number of trials comparing each pair of drug agents were in the direct ratio. The size of the node is proportional to the number of participants and presents the sample size. BNP, brain natriuretic peptide.

We assessed the efficacy of five different interventions including intravenous saline, nitroglycerin, BNP, usual-dose nicorandil, double-dose nicorandil for preventing CIN. The network of comparisons for CIN occurrence and the change of SCr level was shown in Fig. 2. These RCTs included intravenous saline (11 trials, 1,560 participants for CIN; 10 trials, 1,431 participants for SCr;), nitroglycerin (1, 59; 1, 59), BNP (4, 734; 5, 800), usual-dose nicorandil (3, 244; 3, 286), double-dose nicorandil (6, 739; 4, 508).

### The incidence of CIN after the procedure

**Figure 3:**
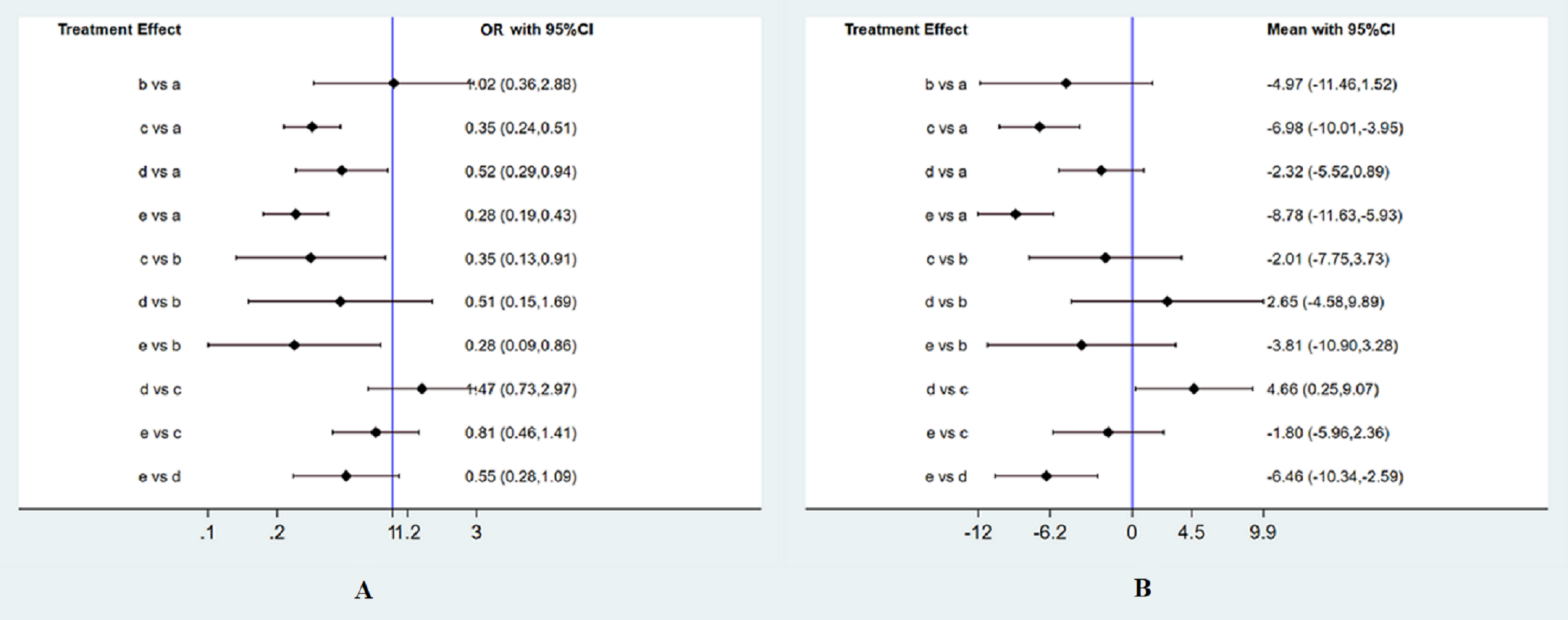
Forest plots of network meta-analysis. (A) Forest plots of network meta-analysis of all trials for decreasing the occurrence of CIN. (B) Forest plots of network meta-analysis of all trials for the efficacy of reducing the change of SCr levels. a, intravenous saline; b, nitroglycerin; c, BNP; d, usual-dose nicorandil; e, double-dose nicorandil. OR, odds ratios.

Totally 12 RCTs including 3,332 participants have evaluated the effect of pharmacological interventions on the incidence of CIN. The result was shown by forest plots (Fig. 3A). Compared with intravenous saline alone, additional administration of nitroglycerin was ineffective for decreasing the CIN incidence (OR, 1.02 95%CI [0.36–2.88] (*P* = 0.973). Unstatistic significance between nitroglycerin and intravenous saline can be viewed the effect on reducing the CIN incidence was equal to each other. On the contrary, BNP and nicorandil much more significantly decreased the CIN than intravenous saline alone (OR of 0.35 95%CI [0.24–0.51] for BNP (*P* < 0.001); 0.52 (0.29, 0.94) for usual-dose nicorandil (*P* = 0.031); 0.28 (0.19, 0.43) for double-dose nicorandil (*P* < 0.001). It demonstrated pharmacological intervention by BNP or nicorandil can effectively reduce the occurrence of CIN.

**Figure 4:**
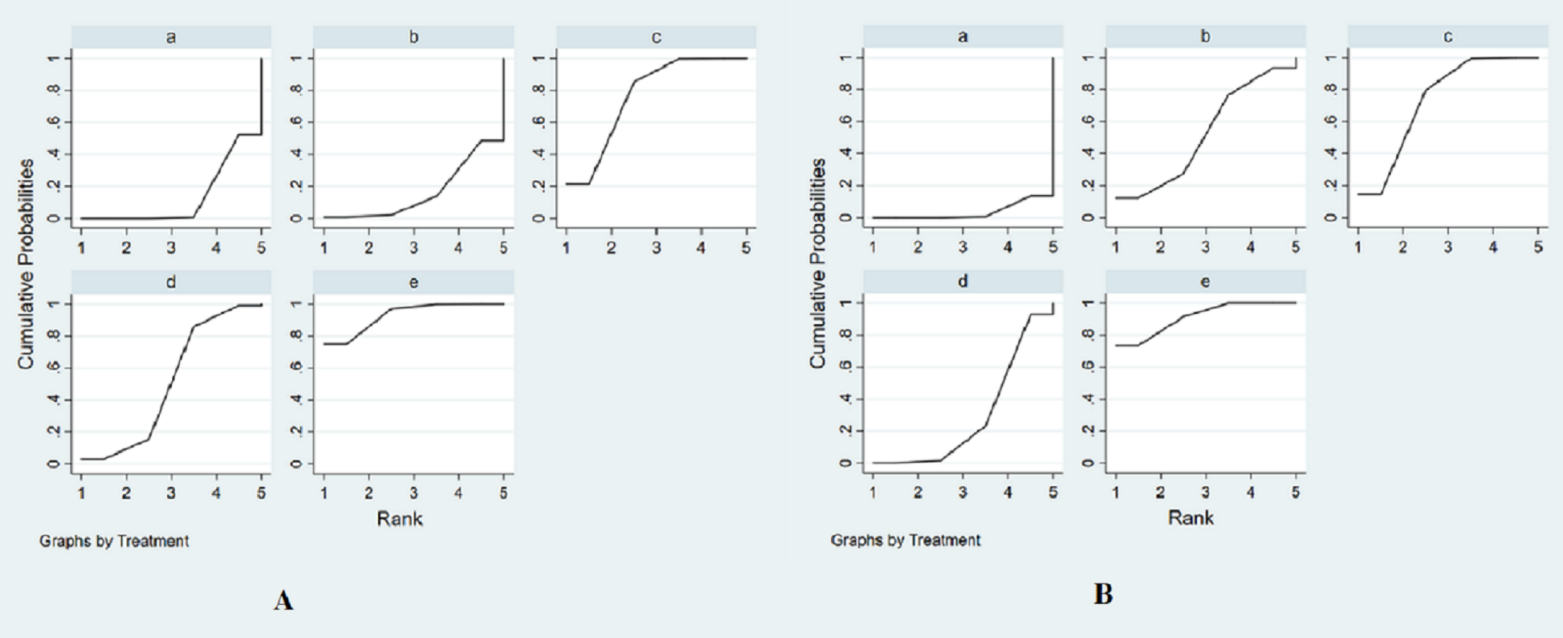
The surface under the cumulative ranking curve (SUCRA) for all interventions in the study. (A) The SUCRA of all agents for decreasing the incidence of CIN. (B) The SUCRA of all drugs for the efficacy of reducing the change of SCr levels. The size of SUCRA is proportional to the efficacy of the treatment. a, intravenous saline; b, nitroglycerin; c, BNP; d, usual-dose nicorandil; e, double-dose nicorandil.

The SUCRA was presented in Fig. 4A. SUCRA was used to rank the efficacy of the drugs interventions in our study. The SUCRA values provided the hierarchy for five interventions that are 13.3, 16.3, 76.6, 50.9, 92.9% of intravenous saline, nitroglycerin, BNP, usual-dose nicorandil, double-dose nicorandil for the incidence of CIN (Table S1). According to the SUCRA, we could find double-dose nicorandil had the best efficacy in the CIN occurrence, followed by BNP and usual-dose nicorandil. It concluded double-dose nicorandil was superior to BNP and usual-dose nicorandil was inferior to BNP in the efficacy of reducing CIN incidence.

**Table 1:** Characteristics of include studies in the network meta-analysis.

*Author /year*	*Size*	*Follow-up*	*Age*	*Study type*	*Interventions (no.)*	*Comparisons*	*Outcomes*	*Measures*	*Risk of bias*
Liu/2014	1000	7days	67y	RCT	0.9%NaCl(1 mL/kg/h) (*n* = 500) BNP(0.005 ug/kg/min) (*n* = 500)	BNP vs 0.9%NaCl	BUN, Scr, eGFR, CIN occurrence	odds ratio Mean ± SD	low risk
Liu/2015	209	1 month	69y	RCT	0.9%NaCl(1 mL/kg/h) (*n* = 103) BNP(0.005 ug/kg/min) (*n* = 106)	BNP vs 0.9%NaCl	Scr, eGFR, CIN occurrence	odds ratio Mean ± SD	low risk
Sun/2015	126	72 h	60y	RCT	0.9%NaCl(1 mL/kg/h) (*n* = 63) BNP(1.5 ug/kg) (*n* = 63)	BNP vs 0.9%NaCl	Scr, CCl, CIN occurrence	odds ratio Mean ± SD	low risk
Xing/2015	116	72 h	64y	RCT	nitroglycerin(20 ug/min) (*n* = 59) BNP(1.5 ug/kg) (*n* = 57)	BNP vs nitroglycerin	Scr, eGFR, Cys-C, CIN occurrence	odds ratio Mean ± SD	low risk
Zhang/2010	149	7days	65y	RCT	0.9%NaCl(0.5-1.5 mL/kg) (*n* = 75) BNP(1.5 ug/kg) (*n* = 74)	BNP vs 0.9%NaCl	Scr, eGFR, CIN occurrence	odds ratio Mean ± SD	low risk
Fan/2016	240	72 h	67y	RCT	0.9%NaCl(1 mL/kg/h) (*n* = 120) nicorandil(30 mg) (*n* = 120)	double-dose nicorandil vs 0.9%NaCl	Scr, eGFR, Cys-C, CIN occurrence	odds ratio Mean ± SD	low risk
Fan/2019	252	72 h	63y	RCT	0.9%NaCl(1 mL/kg/h) (*n* = 125) nicorandil(30 mg) (*n* = 127)	double-dose nicorandil vs 0.9%NaCl	Scr, eGFR, Cys-C, CIN occurrence	odds ratio Mean ± SD	low risk
Iranirad/2017	128	72 h	61y	RCT	0.9%NaCl(1 mL/kg/h) (*n* = 64) nicorandil(10 mg) (*n* = 64)	usual-dose nicorandil vs 0.9%NaCl	Scr, eGFR, CIN occurrence	odds ratio Mean ± SD	low risk
Ko/2013 NCT01103336	166	48 h	71y	RCT	0.9%NaCl(100 mL) (*n* = 85) nicorandil(12 mg) (*n* = 81)	usual-dose nicorandil vs 0.9%NaCl	Scr, eGFR, CIN occurrence	odds ratio Mean ± SD	low risk
Nawa/2015 UMIN000008544	213	1 month	70y	RCT	0.9%NaCl(1.1 mL/kg/h) (*n* = 107) nicorandil(48 mg) (*n* = 106)	double-dose nicorandil vs 0.9%NaCl	Scr, eGFR, Cys-C, CIN occurrence	odds ratio Mean ± SD	low risk
Zeng/2019	330	48 h	66y	RCT	0.9%NaCl(1.1 mL/kg/h) (*n* = 112) usual-dose nicorandil(15 mg) (*n* = 107) double-dose nicorandil(30 mg) (*n* = 111)	usual-dose nicorandil vs 0.9%NaCl double-dose nicorandil vs 0.9%NaCl	BUN, Scr, eGFR, Cys-C,CIN occurrence	odds ratio Mean ± SD	low risk
Zhang, MD/2019	250	72 h	67y	RCT	0.9%NaCl(1.0 mL/kg/h) (*n* = 125) nicorandil(30 mg) (*n* = 125)	double-dose nicorandil vs 0.9%NaCl	BUN, Scr, crCl CIN occurrence	odds ratio Mean ± SD	low risk
Zhang/2019	300	72 h	67y	RCT	0.9%NaCl(1.0 mL/kg/h) (*n* = 150) nicorandil(30 mg) (*n* = 150)	double-dose nicorandil vs 0.9%NaCl	BUN, Scr, Cys-C CIN occurrence	odds ratio Mean ± SD	low risk

**Notes.**

BUN Blood urea nitrogen Scr Serum creatinine eGFR Estimated glomerular filtration rate CIN Contrast-induced nephropathy Cys-C Cystatin C RCT randomized controlled trial SD standard deviation BNP brain natriuretic peptide

**Table 2:** Baseline characteristics of studies included population.

Author /year	Liu/2014		Liu/2015		Sun/2015		Xing/2015		Zhang/2010	
Characteristic	0.9%NaCl	BNP	0.9%NaCl	BNP	0.9%NaCl	BNP	nitroglycerin	BNP	0.9%NaCl	BNP
Number	500	500	103	106	63	63	59	57	75	74
Age- years ± SD	65 ± 8.7	68 ± 9.2	69.8 ± 6.7	67.6 ± 7.2	60.37 ± 9.26	59.35 ± 9.01	58.64 ± 11.51	58.91 ± 9.81	67.27 ± 7.07	65.39 ± 7.51
Male (%)	337(67.4)	347(69.2)	63(61.2%)	70(66.0%)	39(61.9)	38(60.3)	40(67.80)	41(71.93)	53(67.7)	52(73.4)
Body mass index	25.2 ± 5.2	23.7 ± 4.5	25.4 ± 4.2	24.9 ± 5	24.1 ± 3.4	23.8 ± 3.7	26.78 ± 3.77	27.16 ± 4.42	NA	NA
Diabetes mellitus(n%)	244(48.8)	256(51.2)	71(68.9%)	76(71.7%)	18(28.6)	13(20.6)	15(25.42)	18(31.58)	18(24)	24(32.4)
Hypertension(n%)	276(55.2)	293(58.6)	59(57.3%)	62(58.5%)	38(60.3)	35(59.32)	31(54.39)	NA	NA	
LVEF (%)	51 ± 4.4	53 ± 4.6	58.4 ± 10.5	61.1 ± 8.2	61.51 ± 2.97	61.81 ± 3.12	47.43 ± 7.20	44.95 ± 7.80	39.67 ± 4.76	39.14 ± 3.87
Drugs										
ACEI/ARB (%)	NA	NA	NA	NA	23 (36.5)	23(36.5)	44(74.58%)	39(68.42%)	59(78.7)	61(81.3)
*β*-block (%)	NA	NA	NA	NA	49(77.8)	44(69.8)	43(72.88%)	48(84.21%)	17(22.7)	21(28.4)
Statin (%)	491(98.2)	480(96)	102(99%)	103(97%)	NA	NA	56(94.92%)	56(98.25%)	NA	NA
Clopidogrel (%)	500(100)	500(100)	NA	NA	NA	NA	48(81.36%)	51(89.47%)	NA	NA
CCB	NA	NA	NA	NA	24(38.1)	16(25.4)	31(52.54%)	23(40.35%)	NA	NA
Aspirin, n (%)	500(100)	500(100)	NA	NA	NA	NA	NA	NA	NA	NA
CAG, n (%)	175(35)	156(32.2)	36(35%)	33(31.1%)	NA	NA	NA	NA	NA	NA
PCI, n (%)	325(65)	344(68.8)	67(65%)	73(68.9%)	NA	NA	NA	NA	NA	NA

Author/year	Fan/2016		Fan/2019		Iranirad/2017		Ko/2013		Nawa/2015	
Characteristic	0.9%NaCl	double-dose nicorandil	0.9%NaCl	double-dose nicorandil	0.9%NaCl	usual-dose nicorandil	0.9%NaCl	usual-dose nicorandil	0.9%NaCl	double-dose nicorandil nicorandil
Number	120	120	125	127	64	64	85	81	107	106
Age- years ± SD	67.37 ± 6.33	66.07 ± 6.37	65.87 ± 17.62	62.25 ± 16.63	57.64 ± 12.42	61.35 ± 11.77	69.1 ± 10.3	70.8 ± 9.6	70.1 ± 8.1	70.4 ± 7.7
Male (%)	95(79.17)	88(73.33)	67(53.60)	76(59.84)	40(62.5%)	39(60.9%)	51(67.1)	53(72.6)	74(78.7)	80(81.6)
Body mass index	22.28 ± 2.98	22.36 ± 2.19	23.78 ± 5.98	24.35 ± 5.87	27.78 ± 4.8	28.43 ± 5.6	24.8 ± 3.7	24.1 ± 3.2	23.5 ± 2.9	23.4 ± 3.4
Diabetes mellitus(n%)	62(51.67)	66(55.00)	75(60)	81(63.78)	26(40.6%)	27(42.2%)	42(55.3)	30(41.1)	NA	NA
Hypertension(n%)	74(61.67)	69(57.50)	62(49.6)	68(53.54)	41(64.1%)	35(54.7%)	61(80.3)	57(78.1)	NA	NA
LVEF (%)	51.15 ± 6.36	50.36 ± 5.29	53.58 ± 12.77	51.39 ± 10.35	49.14 ± 5.8	48.87 ± 6.8	NA	NA	NA	NA
Drugs										
ACEI/ARB (%)	47(39.17)	56(46.67)	44(35.20)	46(36.22)	NA	NA	43(56.6)	47(64.4)	10(10.6)	(4.1)
*β*-block (%)	94(78.33)	101(84.17)	53(42.40)	61(48.03)	NA	NA	35(46.1)	2(57.5)	23(23.4)	35(35.7)
Statin (%)	112(93.33)	110(91.67)	83(66.40)	89(70.08)	NA	NA	43(56.6)	37(50.7)	NA	NA
Clopidogrel (%)	NA	NA	79(63.20)	83(65.35)	NA	NA	NA	NA	NA	NA
CCB	34(28.33)	28(23.33)	56(44.80)	50(39.37)	NA	NA	36(47.4)	34(46.6)	NA	NA
Aspirin, n (%)	NA	NA	101(80.80)	108(85.04)	NA	NA	NA	NA	NA	NA
CAG, n (%)	75(62.50)	68(56.67)	NA	NA	NA	NA	NA	NA	NA	NA
PCI, n (%)	27(22.50)	31(25.83)	NA	NA	NA	NA	NA	NA	NA	NA

Author/year	Zhang/2019		MD, Zhang/2019		Zeng/2019		
Characteristic	0.9%NaCl	double-dose nicorandil	0.9%NaCl	double-dose nicorandil	0.9%NaCl	usual-dose nicorandil	double-dose nicorandil
Number	125	125	150	150	1112	107	111
Age- years ± SD	67.11 ± 7.19	67.25 ± 6.42	67.0 ± 7.2	67.4 ± 6.6	66.69 ± 7.33	67.09 ± 6.85	65.37 ± 7.19
Male (%)	114(76.0)	118(78.7)	89(71.2)	93(74.4)	67(39.8)	73(68.2)	78(70.2)
Body mass index	25.10 ± 2.02	24.80 ± 2.17	25.1 ± 2.0	24.9 ± 2.2	24.60 ± 3.34	24.85 ± 2.63	24.67 ± 3.10
Diabetes mellitus(n%)	NA	NA	29(23.2)	24(19.2)	18(16.1)	21(19.6)	19(17.1)
Hypertension(n%)	71(47.3)	69(46.0)	NA	NA	59(52.7)	69(64.5)	42(37.8)
LVEF (%)	60.10 ± 6.88	60.11 ± 7.77	NA	NA	9(8.0)	5(4.7)	13(11.7)
Drugs							
ACEI/ARB (%)	132(88.0)	134(89.3)	111(88.8)	111(88.8)	46(41.1)	56(52.3)	56(50.5)
	118(78.7)	116(77.3)	111(88.8)	112(89.6)		
	139(92.7)	136(90.7)	NA	NA			
*β*-block (%)					93(83.8)	80(74.8)	81(73.0)
Statin (%)					NA	NA	NA
Clopidogrel (%)	150(100)	150(100)	NA	NA	112(100)	107(100)	111(100)
CCB	NA	NA	21(16.8)	16(12.8)	NA	NA	NA
Aspirin, n (%)	150(100)	150(100)	NA	NA	112(100)	107(100)	111(100)
CAG, n (%)	NA	NA	NA	NA	NA	NA	NA
PCI, n (%)	NA	NA	NA	NA	NA	NA	NA

**Notes.**

BNP brain natriuretic peptide SD standard deviation LVEF left ventricular ejection fraction ACEI angiotension converting enzyme inhibitors ARB angiotensin receptor blocker CCB calcium channel blockers CAG coronary angiography PCI percutaneous coronary intervention

### Efficacy of SCr level after the procedure

Related to the change of the SCr level, 11 RCTs including 3,084 patients were available to the network meta-analysis. The result was shown by forest plots (Fig. 3B). Although administration of nitroglycerin or usual-dose nicorandil decreased the SCr levels with MD (−4.97, [−11.46, 1.52] (*P* = 0.133) for nitroglycerin and −2.32, [−5.52, 0.89] (*P* = 0.156) for usual-dose nicorandil), there were no statistical differences for nitroglycerin and usual-dose nicorandil compared with intravenous saline. We can confirm that additional administration of nitroglycerin or usual-dose nicorandil except for hydration can’t significantly decrease the SCr level in PCI or CAG. For BNP and double-dose nicorandil, we discovered these two interventions could significantly reduce the SCr level with MD (−6.98, [−10.01, −3.95] (*P* < 0.001) for BNP, −8.78, [−11.63, −5.93] (*P* < 0.001) for double-dose nicorandil).

The SUCRA values provide the hierarchy for five interventions that are 3.5%, 52.4%, 73.4%, 29.5%, 91.2% of intravenous saline, nitroglycerin, BNP, usual-dose nicorandil, double-dose nicorandil for reducing the change of SCr levels (Fig. 4B and Table S2). We concluded BNP and double-dose nicorandil can significantly decrease the SCr level in PCI or CAG. It is not recommended to apply nitroglycerin or usual-dose nicorandil to prevent CIN because of ineffectiveness in reducing SCr level.

### NWA of different courses of double-dose nicorandil intervention

We divided the RCTs of double-dose nicorandil into two groups according to the treatment course for comparing the efficacy in reducing the CIN incidence and the change of SCr level. One group administrated double-dose nicorandil for the course of less than or equal to 24 h, the other group used double-dose nicorandil for 4–5 days. Five RCTs were included in the network of analyzing the CIN incidence and four RCTs were available to discuss the change of SCr level. We can discovered the CIN incidence and the change of SCr level in a group of 4–5 days course were more than a group of less than or equal to 24 h course by OR and MD (1.48, [0.63−3.46]; 2.48, [−1.96, 6.91]) (Fig. S2).

The SUCRA values provided the hierarchy for two-course groups that are 90.7, 59.3% for the group of less than or equal to 24 h course and a group of 4–5 days course in the CIN incidence and are 93.5%, 56.5% for the group of less than or equal to 24 h course and a group of 4–5 days course in the change of SCr level (Fig. S3, Tables S3 and S4). It demonstrated the efficacy of preventing CIN occurrence in a group of less than or equal to 24 h course was better than a group of 4–5 days course before and after the procedure.

### Heterogeneity and inconsistency assessment

All RCTs were tested for the inconsistency assessed by global inconsistency, loop-specific and node-splitting approach between direct and indirect evidence. In global inconsistency, *P* = 0.609 and *P* = 0.797 (>0.05) in the analysis of CIN and SCr level were demonstrated there was no statistical significance about heterogeneity among the evidence. For the loop-specific approach, the IF is 0.06 (95%CI [0.00–1.36]) indicating the treatment effect from direct and indirect evidence are in agreement (Fig. S4). For the node-splitting approach, the results were presented in Tables S5 and S6. *P*-value>0.05 indicated no inconsistency among the direct and indirect comparisons.

### Small-study effect analysis

**Figure 5:**
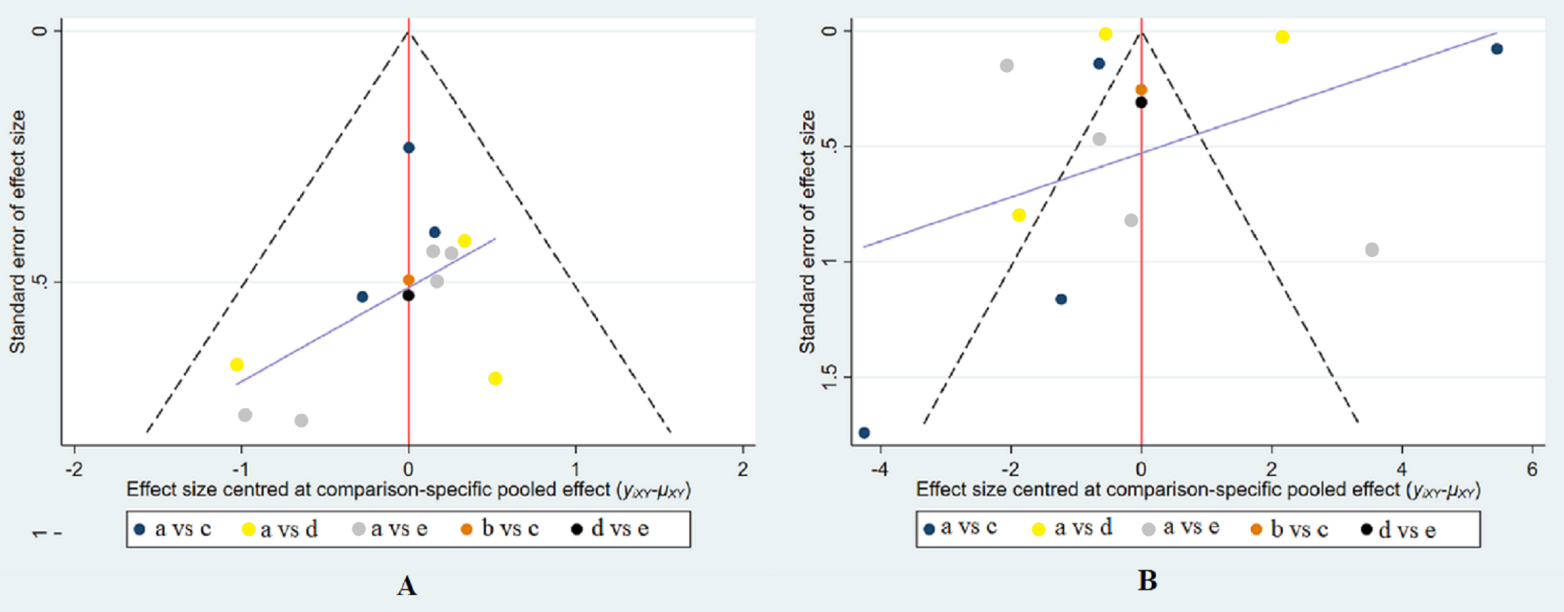
Funnel plot of the treatment in the study. (A) Funnel plot for the assessment of the occurrence of CIN. (B) Funnel plot for the efficacy of reducing the change of SCr levels. The red line represents the null hypothesis that there is no significant difference between the study-specific effect sizes and the respective comparison-specific pooled effect estimates. The purple line is the regression line. Different colors correspond to different comparisons. a, intravenous saline; b, nitroglycerin; c, BNP; d, usual-dose nicorandil; e, double-dose nicorandil.

The results of the comparison-adjusted funnel plots indicated that there may not be small-study effects for efficacy (Fig. 5).

### Clusterank analysis

Multi-purpose arrangement of efficacy for five interventions and two different treatment courses on CIN incidence and SCr level was conducted by clustering analysis (Fig. S5). The best effect intervention was 30 mg daily nicorandil administrated from 12 h before to 12 h after the procedure, followed by BNP (1.5 ug/kg) for 24 h before the procedure.

## Discussion

There is a high risk of the CIN occurrence caused by the administration of contrast agents for patients undergoing CAG and PCI. Periprocedural hydration is the most common method for intervention the incidence of CIN in clinical practical application. For patients with non-dehydration, 500 mL of water was suggested to drink before the contrast examination. In addition, within 24 h contrast exposure administrating 2,500 mL of intravenous saline to sustain a urine generation rate over 1 ml/kg/h (Zhang, Lu & Wang, 2020). This method is effective for preventing the incidence of CIN. However, how much the volume of hydration is sufficient to effectively decrease the incidence of CIN hasn’t been standardized. In addition, the fluids in periprocedural hydration may aggravate disease conditions for patients with heart failure or edema and increase arrhythmias and short-term death risk in the high-risk patient (Nijssen et al., 2019; Nijssen et al., 2020). Therefore, researchers effort to study the treatment of various pharmacological agents in preventing the incidence of CIN. It indicated that compared with intravenous saline alone, pharmacological agents intervention has better benefits to reduce the occurrence of CIN.

Currently, more recent interventions about prostaglandin analogues and BNP analogues are confirmed that could prevent the incidence of CIN. It proved BNP have diuretic and natriuretic action by increasing glomerular filtration rate(GFR) (Potter et al., 2009). This action makes it improving renal hemodynamics and tubular function (Holmes et al., 1993). According to this pharmacological action, several studies have been performed to use BNP to reduce the incidence of CIN in patients undergoing PCI or CAG. Nicorandil is a combination of nicotinamide vitamins and nitrates improving blood flow by opening ATP-sensitive potassium channels and cytoplasmic guanosine cyclase in the kidneys (Shimizu et al., 2011; Fan et al., 2016b). It was shown effective in reducing the incidence of CIN. Whereas there is rare guideline recommend them. One reason probably is inadequate study data could determine the effect of prostaglandin analogues and BNP analogues for preventing the CIN. This study is the first network meta-analysis to specifically evaluate the efficacy of nicorandil (prostaglandin analogues) and BNP for preventing the incidence of CIN after PCI and CAG procedures.

In our study, we made some observations from the evidence of 13 RCTs with 3,462 patients. First, pharmacological agents of BNP and double-dose nicorandil combined with intravenous saline were identified to be beneficial additionally to reduce the occurrence of CIN and the change of SCr levels at 48 h after PCI and CAG procedure than intravenous saline alone. Nitroglycerine and usual-dose nicorandil were similar to the intravenous saline alone on the efficacy of preventing CIN. It suggested that the current evidence supports the clinical application of BNP and double-dose nicorandil in PCI and CAG. Additional administration of nitroglycerin and usual-dose nicorandil have no obvious effect on preventing the CIN occurrence. Second, between double-dose nicorandil and BNP, double-dose nicorandil had the higher SUCRA ranking in reducing CIN occurrence and SCr levels than BNP. These findings demonstrated double-dose nicorandil has better efficacy than BNP for reducing CIN occurrence and the change of SCr levels. However, for consideration of the adverse drug reaction, we supposed BNP is more suitable to prevent the incidence of CIN than double-dose nicorandil. It indicated that more studies can be performed to explore the potential of BNP in reducing the incidence of CIN in the future. Third, the treatment course of less than or equal to 24 h of double-dose nicorandil performed better efficacy than the course of 4–5 days before and after the procedure.

The pathophysiology of CIN is may related to the direct nephrotoxic effects and hemodynamic changes induced by contrast agents. Contrast agents have direct cytotoxic effects on renal tubular epithelial cells and vascular endothelial cells. It could increase the level of endothelin and adenosine and decrease the release of NO and prostaglandins that trigger medullary ischemia and decline GFR in the kidney (Dugbartey & Redington, 2018). In addition, the administration of contrast agents during PCI or CAG could increase the resistance of renal vascular representing sustained vasoconstriction and decrease renal blood flow. The accumulation of contrast agents could create an osmotic environment that induces cellular apoptosis (McCullough et al., 2016). In the condition of overpressure and volume expansion, BNP is released from the membrane granules of cardiomyocytes. The contrast medium can be diluted and excreted by the effect of BNP in increasing diuresis and natriuresis. BNP also could increases GFR by dilating glomerular afferent arteries and constricting the efferent arteries. Nicorandil as prostaglandin analogues increases the renal blood flow by improving the release of nitric oxide and alleviates the inflammatory reaction by antagonizing the production of intracellular oxygen free radicals. The results of our study proved BNP and double-dose nicorandil could prevent the incidence of CIN. Although there is a difference in the mechanism of reducing the CIN between BNP and nicorandil, they all perform an important role in improving renal ischemia. Besides, BNP can aggravates the excretion of contrast agents and nicorandil presents relief of inflammatory reaction. Although this NMA demonstrated double-dose nicorandil presents better action than BNP, we considered it attributes to the reason for increased dose. Therefore, we supported BNP is more suitable to prevent CIN incidence than nicorandil for consideration of adverse drug reactions.

Our results presented a statistically significant reduction of the occurrence of CIN and the change of SCr levels by pharmacological intervention from RCTs. The previous meta-analysis made by Wei et al. (2016b) summarized the incidence of CIN after intervention with BNP from five RCTs with 1,441 patients but was limited to analysis the SCr level change. Past meta-analysis lack a comparison study in treatment effect and intervention dose between BNP and nicorandil for preventing CIN incidence. Compared with these previous reports, there are several advantages to consider in our analysis. First, our study compared the intervention efficacy of BNP and nicorandil for CIN prevention and analyzed the SCr levels change which the previous study hasn’t researched. Second, we made a dose–effect relationship and treatment course comparison for nicorandil. It’s important for using pharmacological intervention during the PCI or CAG by appropriate dosage and course in clinical practice application. In a previous study, the efficacy comparison of different dose drugs and different treatment courses on reducing the incidence of CIN and the change of SCr levels hasn’t been considered. In this work, in the process of analyzing the RCTs of nicorandil, we found the treatment course of nicorandil is different among trails. Based on the efficacy of double-dose nicorandil in preventing CIN, we analyzed whether the treatment course influent the efficacy of double-dose nicorandil. We divided it into two different kinds of the treatment course of double-dose nicorandil. One course is less than or equal to 24 h before and after the procedure, the other course is 4-5 days before and after the procedure. To our surprise, the shorter the course of treatment, the better the treatment effect in decreasing CIN and SCr levels. It was recommended when nicorandil was applied to prevent CIN, 30 mg daily of nicorandil should be administrated from 12 h before to 12 h after the procedure. However, there are some limitations to this study. First, a small number of trials with insufficient participants may affect the accuracy of evaluating the treatment effect. Second, the time of diagnosing the CIN after PCI and CAG varied among studies. Therefore, we analyzed one outcome of CIN incidence by odds ratio according to the result of the included study report. Third, the relationship between CIN and clinical consequences was not investigated because of insufficient data.

## Conclusion

This study is the first network meta-analysis to compare the efficacy of BNP, nicorandil, nitroglycerine, and intravenous saline in preventing the occurrence of CIN. Compared with intravenous saline alone, combined with BNP or double-dose nicorandil (30 mg) could prevent CIN incidence in PCI and CAG. Based on direct and indirect comparison, SUCRA ranking indicates 30 mg daily nicorandil performs better efficacy than BNP (1.5 ug/kg) for 24 h in reducing the SCr levels and CIN incidence. The best course of 30 mg nicorandil is from 12 h before to 12 h after the procedure.

##  Supplemental Information

10.7717/peerj.12975/supp-1 Supplemental Information 1 Raw data for efficacy analysis of drug interventions on the CIN incidence and SCr level change and efficacy comparison of two kinds of the treatment course of double-dose nicorandil Supplemental file 1 is the raw data for efficacy analysis of drug interventions on the CIN incidence and SCr level change and efficacy comparison of two kinds of the treatment course of double-dose nicorandil. In this file, we present the author name of all included studies, the number of participants, trial groups, and outcome of groups. Sheet 1: the raw datafor efficacy analysis of drug interventions on the CIN incidence; Sheet 2: the raw datafor efficacy analysis of drug interventions on the SCr level change; Sheet 3: the raw data for efficacy comparison of two kinds of treatment course of double-dose nicorandil on the CIN incidence; Sheet 4: the raw data for efficacy comparison of two kinds of the treatment course of double-dose nicorandil on the SCr level change;

10.7717/peerj.12975/supp-2 Supplemental Information 2 Primary figure of manuscript Supplemental file 2 is the primary figure of my manuscript. Figure 1. Risk of bias assessment; Fig. 2. Forest plots of two kinds treatment courses of double-dose nicorandil; Fig. 3 The surface under the cumulative ranking curve (SUCRA) for two kinds of treatment courses of double-dose nicorandil in the study; Fig. 4 Loop-specific approach of included study; Fig. 5 Clustering analysis of five interventions and two kinds of treatment courses of double-dose nicorandil.

10.7717/peerj.12975/supp-3 Supplemental Information 3 Primary table of manuscript Supplemental file 3 is the primary table of my manuscript. Table 1 SUCRA analysis for the CIN incidence; Table 2 SUCRA analysis for the outcome Scr levels; Table 3 SUCRA analysis of two kinds treatment course of double-dose nicorandil in the CIN; Table 4 SUCRA analysis of two kinds treatment course of double-dose nicorandil in the SCr; Table 5 node-splitting approach for inconsistency assessment of all comparisons in the CIN; Table 6 node-splitting approach for inconsistency assessment of all comparisons in the SCr level.

10.7717/peerj.12975/supp-4 Supplemental Information 4 PRISMA checklist

10.7717/peerj.12975/supp-5 Supplemental Information 5 Meta-Analysis Rationale

## Abbreviations

NMA: network meta-analysis
BNP: brain natriuretic peptide
CIN: contrast-induced nephropathy
PCI: percutaneous coronary intervention
CAG: coronary angiography
MD: mean differences
95%CI: 95% confidence interval
OR: odds ratio
RCT: randomized controlled trial
PRISMA: Preferred Reporting Items for Systematic Reviews and Meta-Analyses
SCr: serum creatinine
SUCRA: surface under the cumulative ranking curve
eGFR: estimated glomerular filtration rate
IF: Inconsistency factor

## Additional Information and Declarations

### Data Availability

The following information was supplied regarding data availability: The raw measurements are available in the Supplementary File.
