# Supplementary material for: Efficacy of brain natriuretic peptide vs. nicorandil in preventing contrast-induced nephropathy: a network meta-analysis
Source: PeerJ. 2022 Feb 23;10:e12975. doi: 10.7717/peerj.12975 (PMC8881910; doi:10.7717/peerj.12975)
Supplement: Supplemental Information 2 — Supplemental file 2 is the primary figure of my manuscript. Figure 1. Risk of bias assessment; Fig. 2. Forest plots of two kinds treatment courses of double-dose nicorandil; Fig. 3 The surface under the cumulative ranking curve (SUCRA) for two kinds of treatment courses of double-dose nicorandil in the study; Fig. 4 Loop-specific approach of included study; Fig. 5 Clustering analysis of five interventions and two kinds of treatment courses of double-dose nicorandil. [file peerj-10-12975-s002.docx]

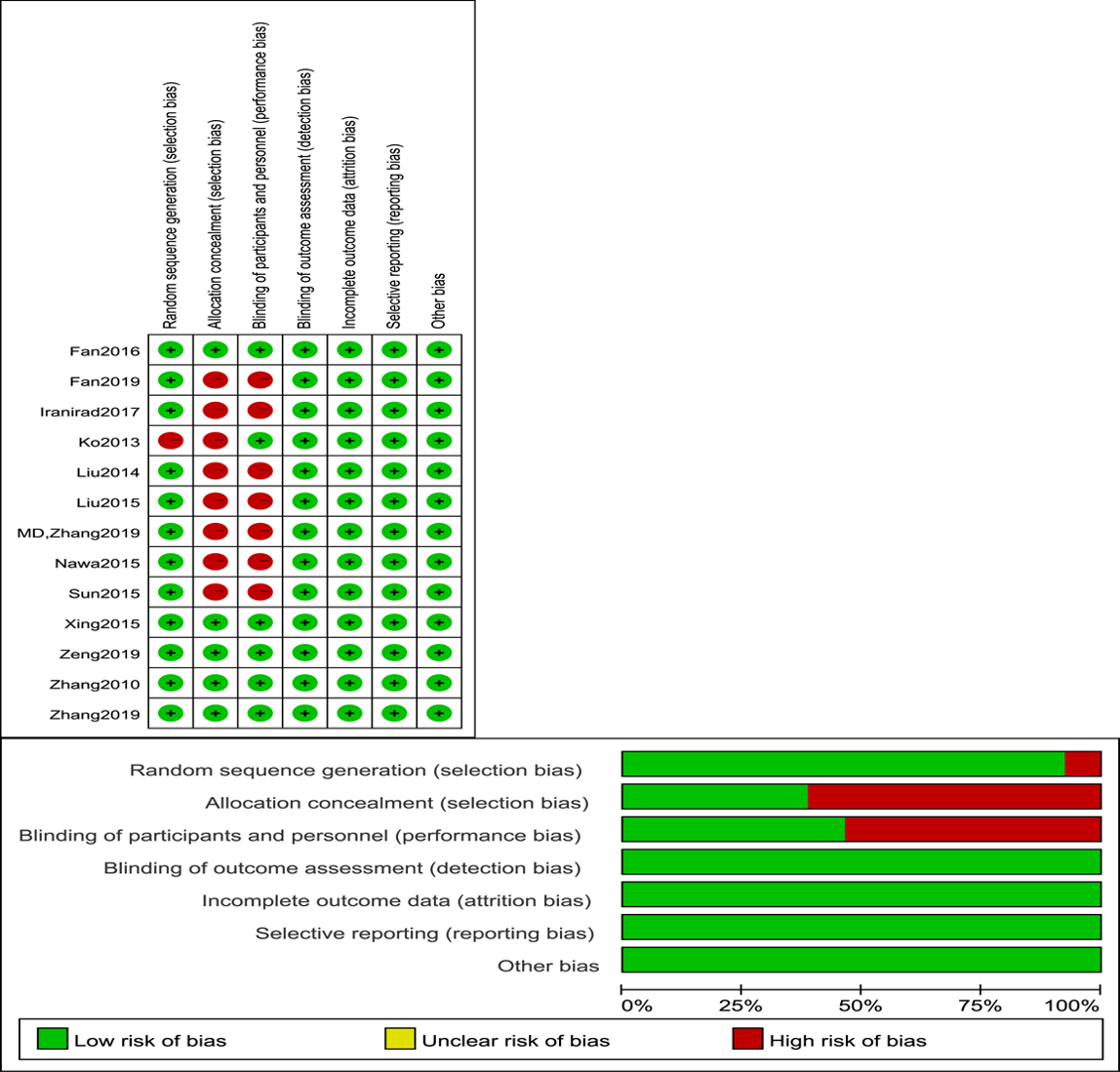


Fig. S1 Risk of bias assessment. The risk bias of included studies was assessed by Cochrane risk-of-bias tool.


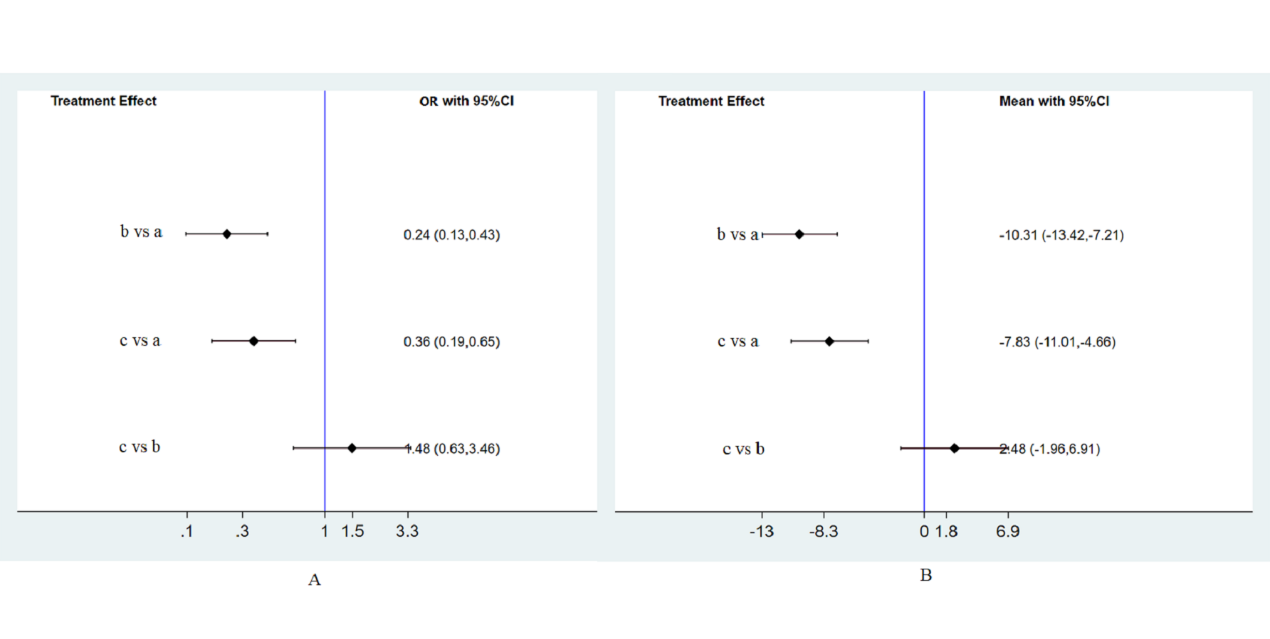


Fig. S2 Forest plots of two kinds treatment courses of double-dose nicorandil. (A) Forest plots of two kinds of treatment courses of double-dose nicorandil for decreasing the occurrence of CIN. (B) Forest plots of network meta-analysis of two kinds of treatment courses of double-dose nicorandil for the efficacy of reducing the change of SCr levels. a=intravenous saline; b=double-dose nicorandil for less than or equal to 24 h; c=double-dose nicorandil for 4-5 days.


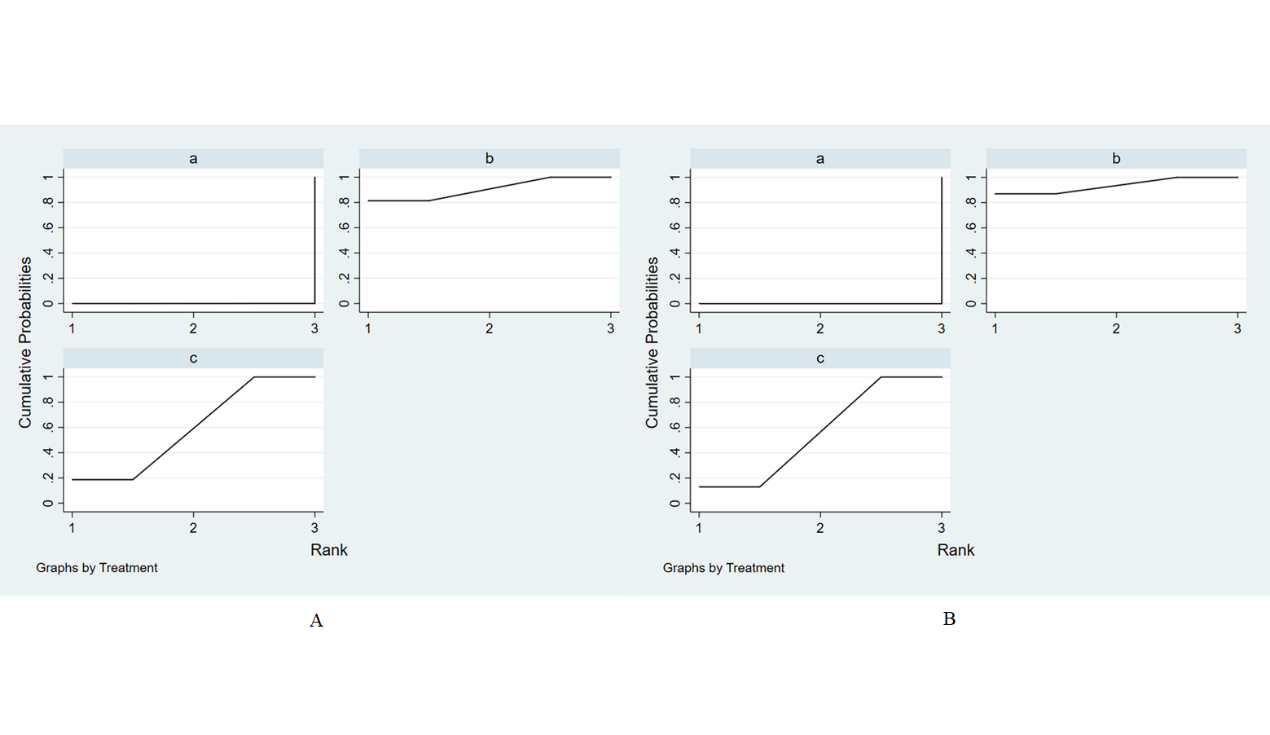


Fig. S3 The surface under the cumulative ranking curve (SUCRA) for two kinds of treatment courses of double-dose nicorandil in the study. (A) The SUCRA of two kinds treatment courses of double-dose nicorandil for decreasing the incidence of CIN. (B) The SUCRA of two kinds treatment courses of double-dose nicorandil for the efficacy of reducing the change of SCr levels. a=intravenous saline; b=double-dose nicorandil for less than or equal to 24 h; c=double-dose nicorandil for 4-5 days.


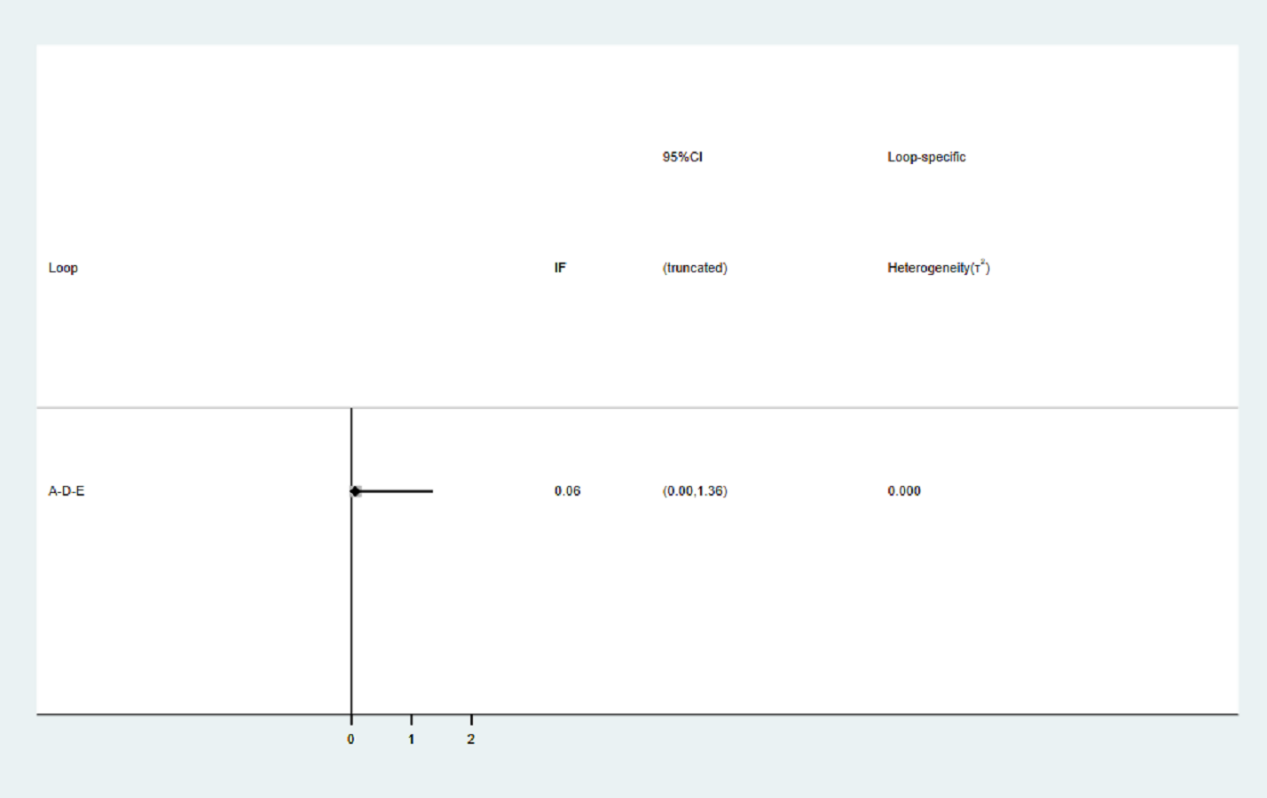


Fig. S4 Loop-specific approach of included study. The loop-specific approach was one method of inconsistency analysis of the included study. A= intravenous saline; D = usual-dose nicorandil; E = double-dose nicorandil.


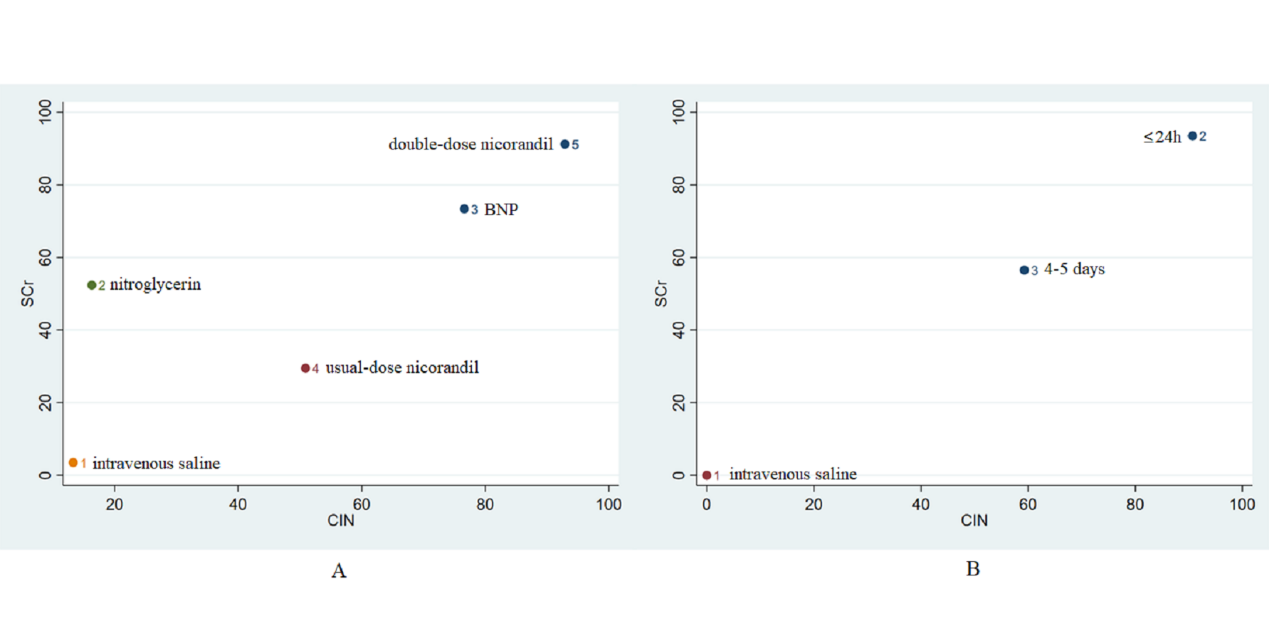


Fig. S5 Clustering analysis of five interventions and two kinds of treatment courses of double-dose nicorandil. (A) Clustering analysis of five interventions for efficacy on the CIN and the change of SCr level. (B) Clustering analysis of two kinds of treatment courses of double-dose nicorandil for efficacy on the CIN and the change of SCr level.
