# Supplementary material for: Efficacy of brain natriuretic peptide vs. nicorandil in preventing contrast-induced nephropathy: a network meta-analysis
Source: PeerJ. 2022 Feb 23;10:e12975. doi: 10.7717/peerj.12975 (PMC8881910; doi:10.7717/peerj.12975)
Supplement: Supplemental Information 3 — Supplemental file 3 is the primary table of my manuscript. Table 1 SUCRA analysis for the CIN incidence; Table 2 SUCRA analysis for the outcome Scr levels; Table 3 SUCRA analysis of two kinds treatment course of double-dose nicorandil in the CIN; Table 4 SUCRA analysis of two kinds treatment course of double-dose nicorandil in the SCr; Table 5 node-splitting approach for inconsistency assessment of all comparisons in the CIN; Table 6 node-splitting approach for inconsistency assessment of all comparisons in the SCr level. [file peerj-10-12975-s003.docx]

**Table S1** SUCRA analysis for the CIN incidence

| Treat | SUCRA | PrBest | Mean Rank |
| --- | --- | --- | --- |
| 0.9%NaCl | 13.3 | 0.0 | 4.5 |
| nitroglycerin | 16.3 | 0.8 | 4.3 |
| BNP | 76.6 | 21.2 | 1.9 |
| usual-dose nicorandil | 50.9 | 3.1 | 3.0 |
| double-dose nicorandil | 92.9 | 74.9 | 1.3 |

BNP: recombinant human brain natriuretic peptide; SUCRA: surface under the cumulative ranking curve

**Table S2** SUCRA analysis for the outcome Scr levels

| Treat | SUCRA | PrBest | Mean Rank |
| --- | --- | --- | --- |
| 0.9%NaCl | 3.5 | 0.0 | 4.9 |
| nitroglycerin | 52.4 | 12.1 | 2.9 |
| BNP | 73.4 | 14.4 | 2.1 |
| usual-dose nicorandil | 29.5 | 0.0 | 3.8 |
| double-dose nicorandil | 91.2 | 73.5 | 1.4 |

BNP: recombinant human brain natriuretic peptide; SUCRA: surface under the cumulative ranking curve

**Table S3** SUCRA analysis of two kinds treatment course of double-dose nicorandil in the CIN

| Treat | SUCRA | PrBest | Mean Rank |
| --- | --- | --- | --- |
| 0.9%NaCl | 0.0 | 0.0 | 3.0 |
| 30mg nicorandil for ≤24h | 90.7 | 81.4 | 1.2 |
| 30mg nicorandil for 4-5 days | 59.3 | 18.6 | 1.8 |

SUCRA: surface under the cumulative ranking curve

**Table S4** SUCRA analysis of two kinds treatment course of double-dose nicorandil in the SCr

| Treat | SUCRA | PrBest | Mean Rank |
| --- | --- | --- | --- |
| 0.9%NaCl | 0.0 | 0.0 | 3.0 |
| 30mg nicorandil for ≤24h | 93.5 | 87.0 | 1.1 |
| 30mg nicorandil for 4-5 days | 56.5 | 13.0 | 1.9 |

SUCRA: surface under the cumulative ranking curve

**Table S5** node-splitting approach for inconsistency assessment of all comparisons in the CIN

| Side | Direct |  | Indirect |  | Difference |  | *P* |
| --- | --- | --- | --- | --- | --- | --- | --- |
|  | Coef | Std | Coef | Std | Coef | Std |  |
| 0.9%NaCl  BNP | -1.044 | 0.188 | -0.688 | 715.472 | -0.428 | 715.472 | 1.000 |
| 0.9%NaCl | -0.644 | 0.315 | -0.829 | 1.053 | 0.185 | 1.084 | 0.864 |
| usual-dose nicorandil |  |  |  |  |  |  |  |
| 0.9%NaCl  double-dose nicorandil | -1.237 | 0.216 | -2.387 | 1.240 | 1.150 | 1.243 | 0.355 |
| nitroglycerin  BNP | -1.062 | 0.495 | -1.895 | 1425.06 | 0.833 | 1425.06 | 1.000 |
| usual-dose nicorandil  double-dose nicorandil | -0.768 | 0.521 | -0.444 | 0.503 | -0.325 | 0.744 | 0.663 |

BNP: recombinant human brain natriuretic peptide; Coef: coefficient; Std: standard deviation; CIN: contrast-induced nephropathy

**Table S6** node-splitting approach for inconsistency assessment of all comparisons in the SCr level

| Side | Direct |  | Indirect |  | Difference |  | *P* |
| --- | --- | --- | --- | --- | --- | --- | --- |
|  | Coef | Std | Coef | Std | Coef | Std |  |
| 0.9%NaCl  BNP | -6.983 | 1.546 | -1.023 | 420.283 | -5.96 | 420.286 | 0.989 |
| 0.9%NaCl | -2.230 | 1.845 | -3.455 | 6.618 | 1.225 | 6.892 | 0.859 |
| usual-dose nicorandil |  |  |  |  |  |  |  |
| 0.9%NaCl  double-dose nicorandil | -9.041 | 1.545 | -3.964 | 6.801 | -5.077 | 6.993 | 0.468 |
| nitroglycerin  BNP | -2.01 | 2.928 | -13.931 | 841.076 | 11.921 | 841.081 | 0.989 |
| usual-dose nicorandil  double-dose nicorandil | -5.247 | 3.086 | -7.467 | 2.815 | 2.220 | 4.177 | 0.595 |

BNP: recombinant human brain natriuretic peptide; Coef: coefficient; Std: standard deviation; SCr: serum creatinine
