# Supplementary material for: Efficacy of brain natriuretic peptide vs. nicorandil in preventing contrast-induced nephropathy: a network meta-analysis
Source: PeerJ. 2022 Feb 23;10:e12975. doi: 10.7717/peerj.12975 (PMC8881910; doi:10.7717/peerj.12975)
Supplement: Supplemental Information 5 [file peerj-10-12975-s005.docx]

Meta-Analysis rationale

Percutaneous coronary intervention(PCI) or coronary angiography(CAG) is common method for diagnosis and treatment of coronary heart disease. However, the use of contrast agents during CAG or PCI usually induce contrast-induced nephropathy(CIN). CIN is a serious complication characterized by worsening of renal function which may lead to water-sodium retention aggravating heart failure and to drug accumulation increasing adverse drug reaction. Nowadays, there is no effective method to therapy CIN. Periprocedural hydration is the most common method for intervention the incidence of CIN in clinical practical application. However, how much the volume of hydration is sufficient to effectively decrease the incidence of CIN hasn’t been standardized. In addition, the fluids in periprocedural hydration may aggravate disease condition for patients with heart failure or edema and increase the risk of arrhythmias and short-term death in high risk patient. Therefore, researches effort to study the therapy of various pharmacological agents in preventing the incidence of CIN.

A large number of randomized controlled trials(RCTs) have demonstrated prostaglandin analogues and BNP analogues are applied to prevent the CIN in the PCI and CAG. Some RCTs showed rhBNP and nicorandil interventions could reduce the incidence of CIN and SCr levels in the PCI and CAG. Whereas there is rare guideline recommend them. One reason probably is inadequate study data could determine the effect of prostaglandin analogues and BNP analogues for preventing the CIN. Therefore a lot of meta-analysis were made to research the efficacy of prostaglandin analogues and BNP analogues for preventing the incidence of CIN in order to develop effect method of reducing CIN for clinical application. Previous meta-analysis made by Xuebiao Wei et al summarized the incidence of CIN after intervention with rhBNP from five RCTs with 1441 patients, but limited to study the SCr level change. Raymond Pranata et al and Bin Yi et al made meta-analysis to demonstrate that nicorandil contributes to a decline in the incidence CIN in patients undergoing CAG/PCI without increasing the major adverse events. However, past meta-analysis lack comparison study in treat effect and intervention dose between rhBNP and nicorandil for preventing CIN incidence. Although they all perform important role on improving the renal ischemia, there is still some difference on the mechanism of reducing the CIN between rhBNP and nicorandil. We supposed it’s necessary to compare the difference in the efficacy between rhBNP and nicorandil for medical workers to choose appropriate preventive therapy. Therefore, we made a NMW to compared the intervention efficacy of rhBNP and nicorandil for CIN prevention and analysed the SCr levels change which previous study hasn’t researched. In addition, we made dose-effect relationship and comparison between rhBNP and nicorandil. In previous meta-analysis, the efficacy comparison of different dose drugs on reducing the incidence of CIN and SCr levels hasn’t been consideration. It’s important for using pharmacological intervention during the PCI or CAG by appropriate dosage in clinical practice application.
